# Wildlife roadkill patterns in a fragmented landscape of the Western Amazon

**DOI:** 10.1002/ece3.6394

**Published:** 2020-06-20

**Authors:** Jonathan Filius, Yntze van der Hoek, Pablo Jarrín‐V, Pim van Hooft

**Affiliations:** ^1^ Wildlife Ecology and Conservation Group Wageningen University & Research Wageningen The Netherlands; ^2^ Universidad Regional Amazónica Ikiam Tena Ecuador; ^3^ The Dian Fossey Gorilla Fund International Musanze Rwanda; ^4^ Grupo de Población y Ambiente Universidad Regional Amazónica Ikiam Tena Ecuador

**Keywords:** Amazon, herpetofauna, hotspots, road ecology, roadkill, spatial patterns

## Abstract

One of the most evident and direct effects of roads on wildlife is the death of animals by vehicle collision. Understanding the spatial patterns behind roadkill helps to plan mitigation measures to reduce the impacts of roads on animal populations. However, although roadkill patterns have been extensively studied in temperate zones, the potential impacts of roads on wildlife in the Neotropics have received less attention and are particularly poorly understood in the Western Amazon. Here, we present the results of a study on roadkill in the Amazon region of Ecuador; a region that is affected by a rapidly increasing development of road infrastructure. Over the course of 50 days, in the wet season between September and November 2017, we searched for road‐killed vertebrates on 15.9 km of roads near the city of Tena, Napo province, for a total of 1,590 surveyed kilometers. We recorded 593 dead specimens, predominantly reptiles (237 specimens, 40%) and amphibians (190, 32%), with birds (102, 17%) and mammals (64, 11%) being less common. Recorded species were assigned to three functional groups, based on their movement behavior and habitat use (“slow,” “intermediate,” and “fast”). Using Ripley's K statistical analyses and 2D HotSpot Identification Analysis, we found multiple distinct spatial clusters or hotspots, where roadkill was particularly frequent. Factors that potentially determined these clusters, and the prevalence of roadkill along road segments in general, differed between functional groups, but often included land cover variables such as native forest and waterbodies, and road characteristics such as speed limit (i.e., positive effect on roadkill frequency). Our study, which provides a first summary of species that are commonly found as roadkill in this part of the Amazon region, contributes to a better understanding of the negative impacts of roads on wildlife and is an important first step toward conservation efforts to mitigate these impacts.

## Graphical abstract

In this study of roadkill in the Amazon region of Ecuador, we found strikingly high numbers of roadkill in a relatively short period, on a short stretch of road. We found multiple distinct spatial clusters or hotspots, where roadkill was particularly frequent. These hotspots were partially explained by land cover variables and road characteristics. Our study, which provides a first summary of species that are commonly found as roadkill in this part of the Amazon region, contributes to a better understanding of the negative impacts of roads on wildlife and is an important first step toward conservation efforts to mitigate these impacts.

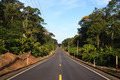

## 1 INTRODUCTION

Understanding the negative effects of roads on wildlife is of increasing importance in a world with rapidly expanding road infrastructure (Laurance et al., 2014). As extensively reviewed by Laurance, Goosem, and Laurance (2009) and Goosem and Marsh (1997), roads lead to the death of organisms by direct collision with vehicles, but roads also create barriers for dispersal, cause habitat change and fragmentation, promote pollution (e.g., noise), alter the microclimate near roads, facilitate the spread of invasive species, and increase human activities such as hunting and deforestation. In turn, these direct effects of roads may alter species behavior, demography, gene flow, and the viability of animal populations (Coffin, 2007; Forman & Alexander, 1998; Jackson & Fahrig, 2011). In recognition of the negative effects of globally expanding road infrastructures, studies on roadkill and potential mitigation measures are increasingly common (Bangs, Bailey, & Portner, 1989; Gibbs & Shriver, 2002; Hels & Buchwald, 2001; Niedziałkowska et al., 2006; Row, Blouin‐Demers, & Weatherhead, 2007; van der Zee, Wiertz, Ter Braak, van Apeldoorn, & Vink, 1992). However, the vast majority of roadkill studies have been conducted in temperate zones, with relatively few published analyses of roadkill in the biodiverse Neotropics (but see Monge, Víquez, & Alvarado, 2013; Payan et al., 2013; Sosa & Schalk, 2016; van Dijck et al., 2013).

Measures to mitigate roadkill, which include educational campaigns and the creation of safe road crossings for wildlife (van der Grift et al., 2013), usually come with high costs and with debates regarding efficiency (van der Ree, Smith, & Grilo, 2015). Therefore, it is important to understand which species are especially vulnerable to roadkill, under which environmental and road conditions roadkill is most prevalent, and how roadkill is spatially distributed. Roadkill does not affect species randomly, as variability in mortality rates between species is linked to species traits; especially those related to movements, microhabitat use, and thermal strategies (González‐Suárez, Zanchetta Ferreira, & Grilo, 2018). For example, species that are large and slow‐moving are likely to be more prone to becoming the victim of vehicle collision, than species that are small and fast‐moving (Forman et al., 2003; González‐Suárez et al., 2018). With regard to species‐specific mortality rates, species that have higher local abundances are more likely to be prone to vehicle collision (Forman et al., 2003).

Roadkill is not randomly distributed along roads, but tend to be most frequent at certain hotspots where environmental and road conditions increase the frequency of affected organisms, or where nearby source habitat creates a steady supply of potential roadkill victims (Barthelmess, 2014; Clevenger, Chruszcz, & Gunson, 2002; Coelho, Teixeira, Colombo, Coelho, & Kindel, 2012; D’Amico, Román, de los Reyes, & Revilla, 2015; Danks & Porter, 2010; Forman & Alexander, 1998; Freitas, Hawbaker, & Metzger, 2010; Hobday & Minstrell, 2008; Kanda, Fuller, & Sievert, 2006). For example, high traffic volumes and the proximity of roads to bodies of open water are associated with high mortality rates (Coelho, Kindel, & Coelho, 2008; Coelho et al., 2012; D’Amico et al., 2015). When we know under which conditions roadkill is most or least prevalent, and when we have identified hotspots of roadkill, we can start attempts at roadkill mitigation, for example by installing wildlife crossing structures such as tunnels, ledges in culverts and overpasses (Clevenger, Chruszcz, & Gunson, 2001; van der Grift et al., 2013).

Although other studies on roadkill, especially those set in the Neotropics (e.g., Coelho et al., 2012; Freitas et al., 2010; Teixeira, Coelho, Esperandio, & Kindel, 2013; Teixeira, Coelho, Esperandio, Rosa Oliveira, et al., 2013), might allow us to derive some generalities, we do not have any actual data on roadkill in the western Amazon, the part of the Amazon where human encroachment through road expansion is highest (Lessmann, Fajardo, Muñoz, & Bonaccorso, 2016; Mena, Lasso, Martinez, & Sampedro, 2017). To provide such initial data, and to start a body of literature which may aid the effective implementation of roadkill mitigation efforts in this biodiverse part of the world, we quantified roadkill incidents on two roads in the Ecuadorean Amazon. For these roads, we determined the prevalence of different species, whether roadkill was spatially clustered, and whether there were any environmental or road characteristics that were likely to influence roadkill prevalence. The intensive history of human presence in our study area, such as habitat conversion and hunting (Sierra, 2000), led us to hypothesize that the landscape is highly defaunated and depleted of mammalian wildlife in particular. Therefore, we expect to find relatively low numbers of mammalian wildlife among our roadkill specimens, as compared to areas with less human pressure (e.g., Coelho et al., 2008 and González‐Gallina, Benítez‐Badillo, Rojas‐Soto, & Hidalgo‐Mihart, 2013), and instead comparatively high numbers of herpetofauna.

Following other studies (e.g., Coelho et al., 2008; Sosa & Schalk, 2016; Trombulak & Frissell, 2000), we made the following five predictions: (a) The majority of our roadkill is represented by slow‐moving species. Although dead specimens will likely be found all along our sampled roads, we also predict that (b) there are locations where roadkill is especially prevalent (hotspots), particularly where native habitat (e.g., native forest, waterbodies, or shrubland) is present close to the road. In addition, we predict that (c) more roadkill is found near wider road stretches with relatively high‐speed limits. We expect that (d) roadkill is especially prevalent where stretches of road have both high‐quality habitat and high‐speed limits (Trombulak & Frissell, 2000). Finally, expanding on the influence of landscape on roadkill prevalence, we predict that (e) roadkill decreases in abundance and diversity as distances to patches of protected high‐quality habitat increases, as we hypothesize that high‐quality native habitat (in our study represented by a protected area) harbors relatively high density and diversity of wildlife that can be subject of vehicle collision; a pattern that we predict to be supported by the lack of wildlife in areas with more human disturbance, such as urban centers, as observed previously by Carvalho and Mira (2011).

## 2 METHODS

### 2.1 Study area

**FIGURE 1:**
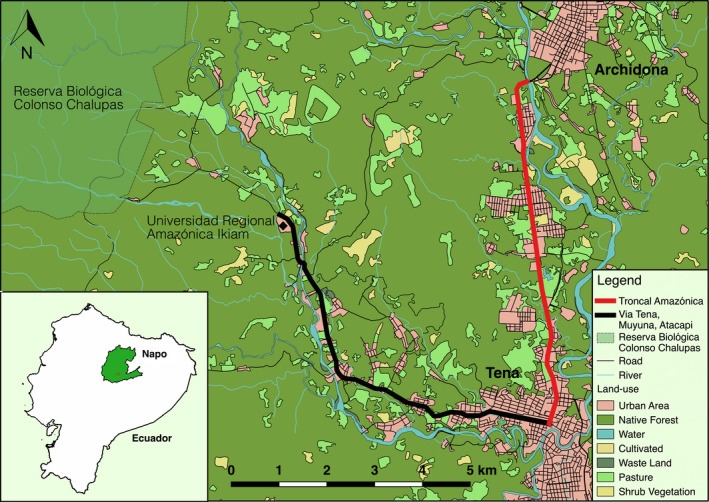
Map of the study area in the canton Tena, Napo province, Ecuador, with the two surveyed roads, Troncal Amazónica (red line) and Via Tena, Muyuna, Atacapi (black line)

We conducted our study in canton Tena (~60,000 inhabitants), the capital of the Amazonian province of Napo, Ecuador (Figure 1; 420 m asl; 0°59′50.1"S, 77°48′45.2"W). The local climate is tropical with an average annual temperature of 23.3°C and annual average precipitation of 4,330 mm (Universidad Regional Amazónica Ikiam meteorological station; van der Hoek, Salagaje, & Ordóñez‐Delgado, 2018). There is little variation in temperatures across months or seasons and rainfall is frequent in every month with no clear dry season, but we note that the year in which we conducted our study (2017) was particularly wet (c. 6,700 mm) and that the months of September–December experienced a higher average rainfall (c. 660 mm) than other months (c. 424 mm) of that year (data derived from Universidad Regional Amazónica Ikiam meteorological station, at meteorologia.ikiam.edu.ec/meteoviewer/).

The study region was formerly comprised of lowland rainforest and foothill evergreen forest, but primary forest is now mainly restricted to the boundaries of Reserva Biológica Colonso Chalupas (RBCC), which was established in 2014. We anticipated that RBCC is rich in vertebrates (van der Hoek et al., 2018), more than the surrounding landscape, and may contain populations of animals which, once leaving the reserve, are vulnerable to roadkill. The remainder of the landscape is currently dominated by a mix of secondary native forests, monoculture agroforestry (e.g., cacao), gardens, cattle pasture, and traditional mixed agroforestry lands known as “chakras” (Torres, Maza, Aguirre, Hinojosa, & Günter, 2015). We are unaware of any accurate account of the history of the forests in the region that would allow us to determine whether forest stands outside the protected area are indeed regrown from previous clearings or disturbances (i.e., secondary forest), though it is clear that the large majority of these forests have seen considerable human intervention. Therefore, we hereafter consider them equivalent to secondary forest.

We selected two roads within the study area that vary in characteristics such as road size and traffic volumes (Figure 1): (a) A 7.43 km long stretch of four‐lane highway, which is known as the Troncal Amazónica (E45), and that connects the towns of Tena and Archidona. This was the largest and most heavily used of the surveyed roads (9,571 vehicles per day in 2016, according to Dirección Municipal de Tránsito Transporte Terrestre y Seguridad Vial (2017)), and was partially paved as early as the 1970s. The speed limit on this road ranges from 50 km/hr to 100 km/hr. (b) A two‐lane road between Tena and Universidad Regional Amazónica Ikiam (Ikiam) of 8.45 km called Vía Tena, Muyuna, Atacapi (VTMA). This road was recently asphalted (in phases between 2011 and 2013) and had considerably less vehicular movement than the E45, although exact traffic rates were unknown. The speed limit on this road ranges from 50 km/hr to 90 km/hr. The landscape along both the E45 and Vía Muyuna consists of a mix of urban area, native secondary forest, pasture, and cultivated areas.

### 2.2 Data collection

Between September and December 2017, we surveyed the two roads for 5 days a week, for a total of 50 days. We surveyed both roads by bicycle with a cycling speed between 10 and 14 km/hr, well below the 20 km/hr that tests show provide high detection probabilities in vehicle‐driven surveys (Collinson, Parker, Bernard, Reilly, & Davies‐Mostert, 2014). Each survey started within 24 hr of the previous survey, with a 2‐day break on Saturday and Sunday, approximately 2 hr after sunrise (~7.30–8.00 a.m.). For logistical reasons, we surveyed the roads one after the other within 3 hr, always in the same order. We collected data from all nondomesticated reptiles, amphibians, birds, and mammals found dead on the roads and recorded GPS coordinates, the probable cause of death, and the location on the road. We took photographs to guide identification and removed roadkill from the road surface to prevent double counting.

To effectively focus conservation efforts on the most vulnerable wildlife, we recognize that the frequency of roadkill, and factors that influence roadkill, must be evaluated for different groups or species separately (Markwith, Evans, Da Cunha, & De Souza, 2020). For the analyses of spatial patterns, we classified the roadkill into three functional groups based on movement speed and habitat use (e.g., fossorial, terrestrial, or aerial). The first group (a) “slow,” consisted of slow‐moving fossorial/terrestrial species (including snakes, amphisbaena, and caecilians), the second group (b) “intermediate,” of intermediate‐moving terrestrial species (including frogs and toads), and the last group (c) “fast,” of fast‐moving aerial species (including birds) (Table S1) (Ciocheti, de Assis, Ribeiro, & Ribeiro, 2017; Markwith et al., 2020). The species belonging to these groups formed the majority of roadkill specimens in our study. Other specimens that did not fit into one of these groups were excluded from further analysis because they would likely cause noise but are shown in Table S1. Mammals were also excluded from the analyses, since most of the recorded specimens were rodents, presumably belonging to the genus *Rattus,* as well as domestic species which we did not consider relevant from a conservational standpoint.

We obtained land cover data for 2012 from the Ministerio de Agricultura, Ganaderia y Pesca (Ministerio de Agricultura Ganaderia y Pesca, 2012). These vector data were based on Landsat raster imagery with a 30‐m resolution. We virtually divided the roads (total 15.9 km) into 159 segments of 100 m. We used QGIS version 2.18.14 (QGIS Development Team, 2014) to calculate the percentage of each of the seven different land cover classes (i.e., urban area, native secondary forest, pasture, shrubland, cultivated land, and waterbody) within rectangular buffers around each segment. Since we did not know what the best spatial scale to relate roadkill to land cover was, we used three different buffer sizes (Langen, Ogden, & Schwarting, 2009; Markwith et al., 2020). The buffers extended 100, 200, and 500 m to both sides of a road segment, covering a total area from 2 to 10 ha. We subsequently determined the centroid of each road segment and calculated the distance of the centroid to the nearest waterbodies (rivers or lakes), the RBCC, and the center of the cities Tena (0°59′43.0″S 77°48′53.9″W) and Archidona (0°54′56.3″S 77°48′33.9″W). As we did not have access to data on vehicular traffic volumes or densities for all roads, we could not include this in our analyses although this is often an important variable that explains variation in roadkill (Forman & Alexander, 1998). Finally, we surveyed the length of all roads on bike and registered the speed limit as posted alongside (ranging from 50 km/hr to 100 km/hr) the road and recorded the number of lanes (2 or 4). We used these factors as additional categorical predictors.

### 2.3 Statistical analyses

We used the heatmap plugin in QGIS (QGIS Development Team, 2014), which uses kernel density estimation, to create a heat map (i.e., a density map) of vertebrate roadkill. We tested for clustering in the spatial distribution of roadkill, using Ripley's K‐statistics in SIRIEMA v2.0 (Coelho, Coelho, Kindel, Texeira, 2014). Ripley's K‐statistic is used to evaluate the dispersion of roadkill events over multiple scales (Cressie, 1993; Newton & Ripley, 1984). It estimates the expected number of random points within a distance *r* of a randomly chosen point along a line and can be used to calculate whether points are randomly distributed along this line or not. We grouped the two roads, as they are sequentially connected, and considered them to form one line for these analyses (i.e., the part of the line from 0 to 7.43 km represents the E45 and the part from 7.43 to 15.88 the VTMA). For all roadkill together, and for the functional groups “slow,” “intermediate,” and “fast” separately, we ran 1,000 simulations with an initial radius of 50 m and radius steps of 50 m (Coelho et al., 2012) and subsequently tested for significance of the spatial homogeneity of our roadkill observations. It is important to identify whether the distribution of roadkill incidents has significant spatial clustering and on which scales they occur, before deriving hotspots. Observed *K*‐values (*K*(*r*)) were subtracted from simulated *K*‐values (*Ks*(*r*)), which in turn resulted in *L*‐statistics (*L*(*r*) = *K*(*r*) − *Ks*(*r*)). *L*(*r*) values above the confidence limits (99%) indicate scales with significant clustering, while values below the limits indicate scales with significant dispersion (Coelho et al., 2014). After the identification of clusters, we identified roadkill hotspots, using the 2D HotSpot Identification Analysis in SIRIEMA v2.0 (Federal et al., 2014). We again used a radius of 50 m and permuted 1,000 simulations, and identified roadkill hotspots as those roadkill intensity values that fell above the upper 95% confidence limits (Coelho et al., 2012).

To analyze the influence of spatial predictors on roadkill intensity, we fitted generalized linear models (GLMs) with a Poisson distribution and log link function, with the glm function in base R (R Core Development Team, 2013), and using the number of roadkill per road segment as a response variable. We centered and standardized all continuous variables by subtracting the mean of each variable from each observation and dividing it by the standard deviation. Before fitting our GLMs, we first calculated Spearman's rank correlation coefficients to identify collinearity between predictor variables following Danks and Porter (2010). We did not find highly correlated variables (for all our variables: *ρ* ≤ |0.70|, Table S4). Our final set of potential predictor variables included: amount (%) of native forest, urban area, pasture, shrubland, cultivated land, and waterbody; distance (km) to water, RBCC and city, and speed limit and number of lanes (as categorical variables).

We first used GLMs with a Poisson distribution and log link function to check for significant relationships between each predictor variable and the response variable (roadkill per road segment), that is, simple univariate linear models with one predictor variable each. Subsequently, we developed a suite of models based on our formulated hypotheses. All hypotheses involving land cover variables were evaluated for the three different buffer sizes. We fitted GLMs models with a combination of predictors that represented these hypotheses and searched for the model with the best fit. As previously mentioned, (2) we hypothesized that the quality of the habitat along the roads influences the number of roadkill; therefore, we fitted a model that included the land cover predictors native forest, shrubland, and waterbody but also a model with all land cover predictors. (3) Another hypothesis was that the road characteristics “speed limit” and “number of lanes” affect roadkill prevalence; thus, we fitted a model with these predictors. (4) We also fitted a model for the combination of hypotheses 2 and 3. Finally, (5) we hypothesized that the distance to high‐quality habitat (i.e., distance to RBCC and waterbodies) or poor‐quality habitat (i.e., distance to the cities Archidona and Tena) would play a significant role in roadkill distribution. A model including all predictors was also assessed.

We calculated model support using Akaike weights (wAICc, with numbers closer to 1 indicating greater support) (Guthery, Burnham, & Anderson, 2003). We estimated model fit using *D* ^2^, the amount of deviance that is accounted for in the model (Guisan & Zimmermann, 2000). Finally, we fitted Pearson residuals against the *x* and *y* coordinates to check whether spatial clustering was statistically problematic. There is no spatial clustering when there is no clustering of positive or negative residuals or clustering of large (absolute) values (Zuur, Ieno, Walker, Saveliev, & Smith, 2009).

## 3 RESULTS

We recorded a total of 593 dead vertebrates (Table S1), the majority of which were reptiles (237 specimens, 40% of the total number of specimens) and amphibians (190, 32%), with birds (102, 17%) and mammals (64, 11%) being less common. Most species belonged to the functional group “slow” (213 specimens) followed by “intermediate” (140) and “fast” (92). We identified 93% of the 237 reptiles to species level and counted a total of 28 species. The amphisbaenian *Amphisbaena bassleri* (58 records), and the snakes *Atractus elaps* (33), *Atractus major* (32), and *Atractus collaris* (29) were the most prevalent among our records. We recorded 190 amphibians, of at least 11 species, the most common of which were *Rhinella marina* (82) and *Rhaebo ecuadorensis* (29). Only 143 of the 190 amphibians could be determined to species level due to a large amount of damage and decay of many of the specimens. Similarly, we were only able to determine the species of 26 out of 102 birds, recording a total of eight species. In addition, we identified six specimens to genus level and determined that these belonged to three different genera not recorded in the aforementioned eight identified avian species. In total, we found at least 11 bird species along our surveyed roads. Fourteen mammals were bats, none of which we were able to determine to species level. Finally, outside the scope of this article on wildlife but noteworthy nonetheless, was the prevalence of domestic species *Canis lupus familiaris* (dog), *Felis catus* (cat), *Gallus gallus domesticus* (chicken), and invasive rodent species *Rattus sp*. (rats).

Over the course of 50 days, we covered a total of 1,590 km of road. Our total detection rate was 0.37 individuals per surveyed km, with an average of 11.68 dead specimens found per day on 15.9 km of road, which implies an average roadkill estimate of 0.75 animals/km/day. The proportion of roadkill per taxon differed between the two roads (*p* < .001, Fisher's exact test). On road E45, amphibians were most prevalent (34% of specimens), whereas reptiles were most frequent on the VTMA (44%). More birds were killed on the E45 than on the VTMA. None of the species recorded is considered globally threatened by the IUCN, but the conservation status of 22 species is yet to be evaluated (IUCN, 2015).

**FIGURE 2:**
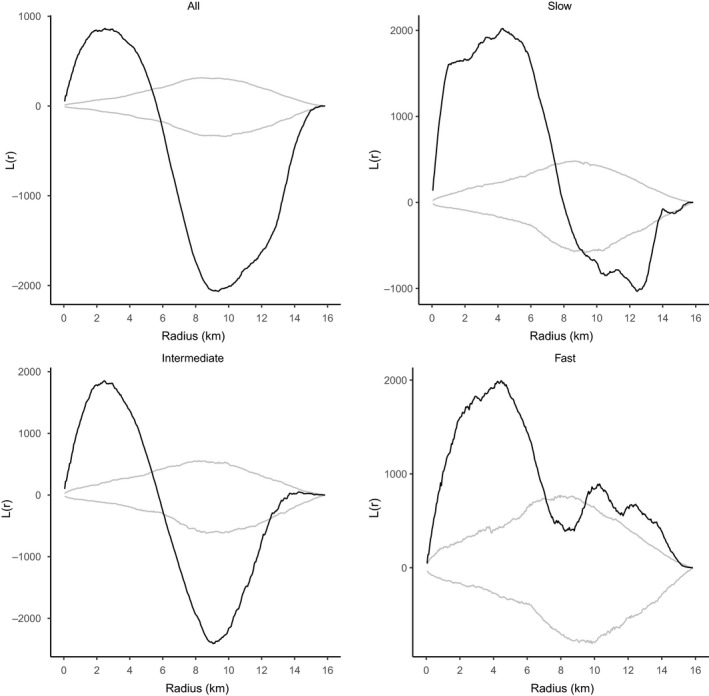
*L*(*r*) statistic (black line) against scale distance (radius), with 99% confidence limits (grey lines) for all vertebrates and per functional group along the two grouped roads E45 and VTMA. The black line represents the observed number of neighbors per roadkill along the road minus the mean expected number of neighbors if the roadkill was distributed randomly. Values above the upper confidence limit (grey line) indicate significant clustering of roadkill

**FIGURE 3:**
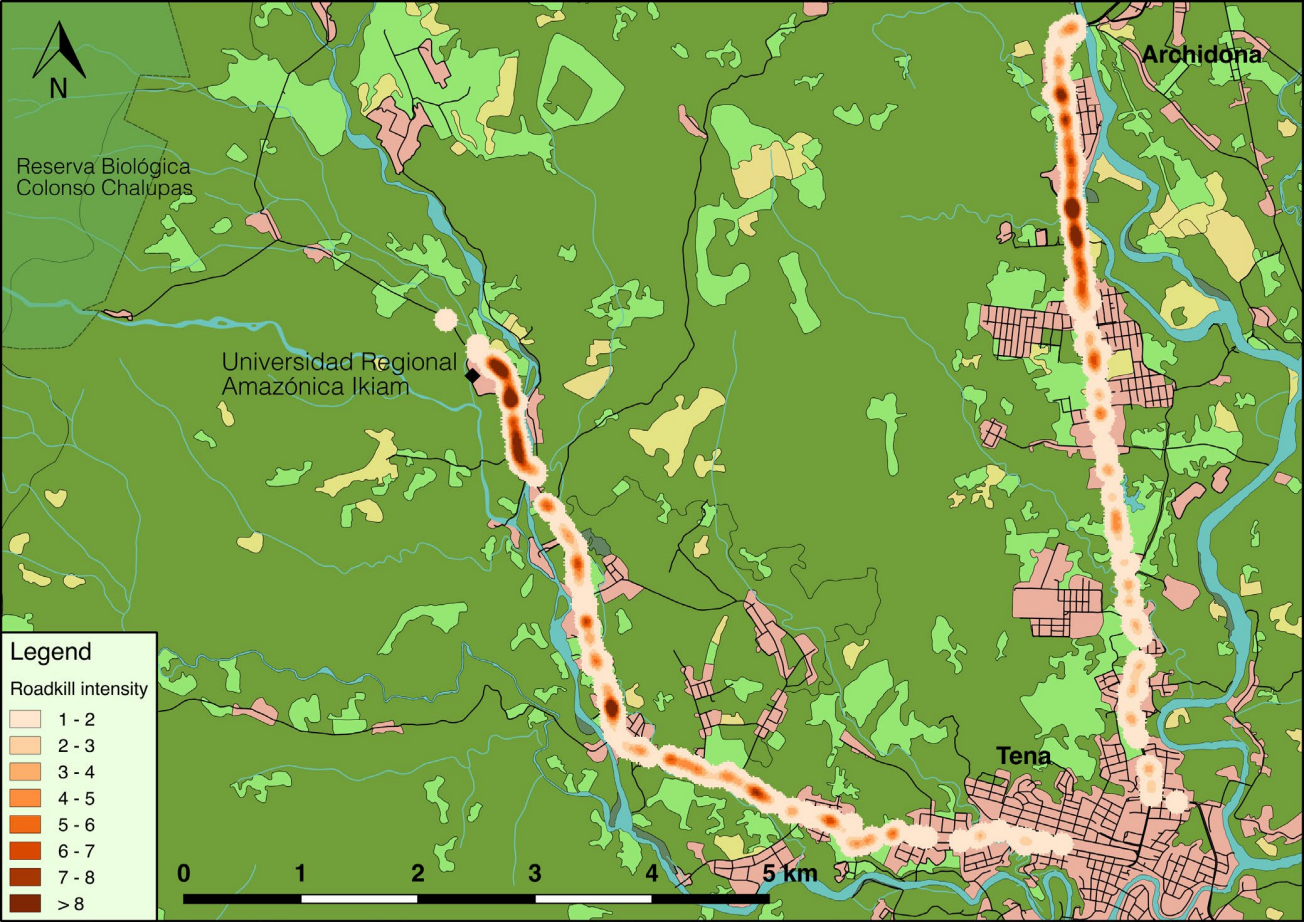
Intensity of roadkill aggregations for all recorded roadkill (roadkill hotspots). The higher the intensity (red color) the higher the aggregation of roadkill

**FIGURE 4:**
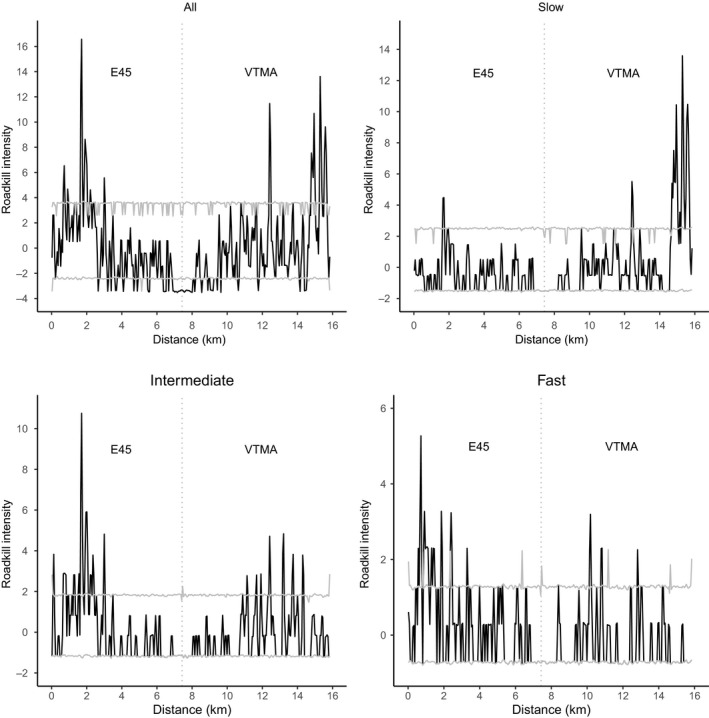
Roadkill intensity of aggregation (black line) and 95% confidence limits (grey lines) for all roadkill and for each functional group along the total 15.9 km of the E45 and VTMA. Values above the upper confidence limit (95%) indicate significant hotspots of mortality

Ripley's *K* statistical analyses showed that clustering of roadkill occurred at scales up to 7.5 km (Figure 2), indicating that roadkill is not randomly distributed along the roads. The 2D Hotspot analysis showed several places along the road where there is a noticeable higher frequency of roadkill and that may be the focus for further efforts to determine priorities in conservation management (Figures 3 and 4). Some sections with roadkill hotspots coincided for the different functional groups, but the groups also showed unique hotspots (Figure 4). We detected hotspots at kilometer 1.8 on E45, at km 12.4 and between km 14.5 and 16 on the VTMA for the “slow” group. The “intermediate” group showed hotspots between km 0 and 3.5 on the E45 and between km 11 and 14.5 on the VTMA. For the “fast” group, we found hotspots between km 0.8 and 2.4 and at km 2.8 on the E45, and at km 10.2, 11.1, and 12.8 on the VTMA.

In the univariate models, where roadkill was only tested against one predictor, most factors were significant on all tested scales (Table S2). As predicted, we found that the distance to RBCC (*p* < .001, *D* ^2^ = 0.303) had a significant positive effect, while the distance to the cities Archidona and Tena (*p* < .001, *D* ^2^ = 0.285) had a significant negative effect on the number of roadkill for species belonging to the group “slow.” Speed limit had a significant positive effect on the number of roadkill for the group “intermediate” (*p* < .001, *D* ^2^ = 0.109) and “fast” (*p* < .001, *D* ^2^ = 0.299) (Table S2). However, model support (*D* ^2^) was weak for all univariate models, indicating that a combination of factors explains the variation in the data better.

The best models (with a Δ AICc ≤ 7; Burnham, Anderson, & Huyvaert, 2011) for “slow” species were those where all predictors were included at buffer sizes of 100 and 500 m, respectively (AICc = 485.2, *D* ^2^ = 0.390 and AICc = 484.22, *D* ^2^ = 0.393) (Table S3). The best‐supported hypothesis for the group “intermediate” included a combination of land cover and road characteristics on a scale of 500 m (AICc = 399.47, *D* ^2^ = 0.241). The best supported models for the group “fast” where those with a combination of land cover and road characteristics on all distance scales (AICc = 343.1, *D* ^2^ = 0.207, AICc = 341.86, *D* ^2^ = 0.214 and AICc = 344.79, *D* ^2^ = 0.199), as well as those where only road characteristics were included (AICc = 342.1), *D* ^2^ = 0.182 . The Pearson residuals plotted against the *x* and *y* coordinates did not indicate problematic spatial clustering in the models.

## 4 DISCUSSION

We show that roadkill is prevalent on the studied roads near Tena and confirmed (a) that relatively slow herpetofauna (i.e., snakes, amphisbaenians, and caecilians) makes up the majority of roadkill in the studied region, and we found (b) that roadkill patterns were nonrandom and centered around hotspots where future mitigation efforts may be most effective or most needed (van der Grift et al., 2013), and recognized that the type of land cover adjacent to roads explained parts of the variation in roadkill distribution, but that (c) road characteristics (e.g., speed limits) were also strong predictors of roadkill. (d) Variation in roadkill patterns was also partially explained by a combination of land cover and road characteristics. Finally, (e) we found that the number of roadkill increased with decreasing distance to RBCC and decreased with increasing distance to the city centers of Tena and Archidona. The importance and significance of each of these factors differed between functional groups. In conclusion, roadkill patterns in the western Amazon show similarities with those observed in other studies (Brum, Santos‐Filho, Canale, & Ignácio, 2018; Coelho et al., 2008; Vargas‐Salinas, Delgado‐Ospina, & López‐Aranda, 2011). However, we found that herpetofauna is affected the most whereas in other studies (Coelho et al., 2008; Miranda et al., 2017; Teixeira, Coelho, Esperandio, & Kindel, 2013; Teixeira, Coelho, Esperandio, Rosa Oliveira, et al., 2013) mammals are often the most affected group.

Differences in vertebrate community composition between our study area and those landscapes surveyed in other studies may have contributed to the relatively high numbers of herpetofauna versus mammals in our study. Mammals are usually found to be the most common group in other studies in the Neotropics (Table 1), with the notable exception of a study conducted in the Central Andes of Colombia which also found particularly high mortality among reptiles and amphibians (De La Ossa‐Nadjar & De la Ossa, 2013), likely reflecting an overall high abundance of mammalian wildlife in these more undisturbed regions. In contrast, mammals in our study area were unlikely to be abundant, and thus a potential victim of roadkill, due to high current and historical levels of human disturbance, including past hunting activities (Sierra, 2000).

**TABLE 1:** Number of roadkill per taxa for eleven different studies conducted in the Neotropics

Study	Reptiles	Amphibians	Birds	Mammals	Total	Roadkill per surveyed km	Length of study
Brum et al. (2018)	11	10	9	135	165	0.035	1 year
Payan et al. (2013)	86	5	59	190	340	0.0034	3 months
Bueno, Sousa, and Freitas (2015)	27	0	92	178	297	–	3 years
Miranda et al. (2017)	55	9	223	826	1,113	–	1 year
De La Ossa‐Nadjar and De la Ossa (2013)	219	253	25	111	608	–	1 year
Cuyckens, Mochi, Vallejos, Perovic, and Biganzoli (2016)	24	0	72	196	293	–	1 year
Coelho et al. (2008)	152	NR	169	548	869	0.028	1 year
da Cunha et al. (2010)	9	NR	34	265	308	0.014	1 year
González‐Gallina et al. (2013)	53	1	65	827	946	–	1 year
Teixeira, Coelho, Esperandio, and Kindel (2013), Teixeira, Coelho, Esperandio, Rosa Oliveira, et al. (2013)	438	NR	743	1,341	2,522	–	3 years
Braz and França (2016)	319	97	213	195	824	0.096	1.5 year
This study	237	190	102	64	634	0.37	3 months

Abbreviation: NR: Not Recorded.

Other reasons and significance for such a contrasting result should be a matter of further studies but are likely to be at least partially influenced by variation in sampling methods. For example, seasonality in activity patterns may influence roadkill patterns and consequently studies may have different outcomes depending on the timing of their sampling (Sosa & Schalk, 2016). In addition, amphibian and reptile mortality could have been underestimated in other studies due to sampling method and effort (Ratton, Secco, & da Rosa, 2014; Teixeira, Coelho, Esperandio, & Kindel, 2013; Teixeira, Coelho, Esperandio, Rosa Oliveira, et al., 2013). In contrast to our sampling by bike, most roadkill studies are done by car, and smaller vertebrates such as frogs and small snakes are therefore more easily missed than mammals, which tend to be larger (D’Anunciação, Lucas, Silva, & Bager, 2013; Santos et al., 2016; Slater, 2002). This could also partially explain the relatively high roadkill per surveyed km in this study compared to estimates by Brum et al. (2018) and Payan et al. (2013). Finally, the persistence time of carcasses is generally lower for reptiles and amphibians than for mammals (Santos, Carvalho, & Mira, 2011), which may also have led to an underestimation of reptile and amphibian mortality in other Neotropical studies, performed with a longer sampling interval (e.g., a week). As we made a more frequent (daily) effort to survey for roadkill, we might have had a higher detection rate of herpetofauna, thus increasing the relative proportion of this group among the roadkill recorded.

In line with other studies, where buffer sizes also were not determined a priori, we found that not all buffer sizes produced the same results (de Freitas et al., 2015; Markwith et al., 2020; Ng, Nielsen, St, & Clair, 2008). The scale that generated the highest quality models varied between functional groups but also between different landscape characteristics. Differences between groups may be caused by differences in habitat preference, mobility, and behavior in relation to landscape characteristics. When pooling species into groups by taxon or traits, intraspecific differences may get lost. In order to target conservation efforts to the most vulnerable species, it is therefore important to identify species that are of higher conservation interest and perform individual analysis on those (Markwith et al., 2020).

We propose that our approach using a combination of large extent, coarse resolution GIS‐derived variables (e.g., native forest cover) and generalized field‐based metrics (e.g., deriving vehicle speeds from speed limits set for a stretch of road) allowed to find particular combinations of suitable habitat and high vehicle speed that lead to the definition of roadkill hotspots. However, we suggest that additional, finer resolution, field‐derived habitat and landscape variables (e.g., related to type and structure of vegetation) could be assessed at—or near, for comparison—these hotspots to allow for a closer examination of the links between road, habitat, and landscape characteristics and the propensity of roadkill of specific species or groups of animals. In addition, as we did not have access to data on traffic density for both roads we were not able to include this in our models. It is, however, an important factor in determining roadkill patterns (Forman & Alexander, 1998) and should be measured and included in future studies.

Despite our efforts to conduct surveys with continuity and accuracy, we acknowledge that there are potential biases in our attempt to estimate the prevalence of roadkill and to determine the factors that influence roadkill frequency. For one, we conducted our study over the course of one part of the year with relatively high amounts of precipitation and do not know if roadkill is equally frequent in other times of the year. Our limited knowledge of the ecology of the species found in our study does not allow us to make precise predictions. However, other studies in the Neotropics have shown that roadkill can be either more (da Cunha, Moreira, & Silva, 2010) or less (Machado, Fontes, Mendes, Moura, & dos RomãO, 2015; Sosa & Schalk, 2016) frequent in the dry as compared to the wet season, a pattern that may be linked to changes in the rates of animal movements in response to food availability. The short period of this study might also have led us to miss so‐called hot moments in the seasonal activity of particular species: These are moments of the year in which species are more vulnerable to be hit by vehicles (e.g., breeding season) (Beaudry, Demaynadier, & Hunter, 2010; Garrah, Danby, Eberhardt, Cunnington, & Mitchell, 2015). We recommend long‐term monitoring that could reveal seasonal patterns as well as changes in roadkill rates over the years due to, for example, ongoing conversion of native forest to other land cover types.

We point out that we surveyed a relatively small area, from which it may prove difficult to extrapolate across the larger region. We made the decision to survey this relatively small region due to the trade‐off between detection accuracy and kilometers surveyed (Santos et al., 2015; Teixeira, Coelho, Esperandio, & Kindel, 2013; Teixeira, Coelho, Esperandio, Rosa Oliveira, et al., 2013). By choosing a bicycle over a vehicle, we were unable to survey long stretches of road but likely increased detection probability and accuracy. However, detection rates were unlikely to be 100%, as dead animals might have gone unnoticed when they were covered by vegetation growing in gullies adjacent to the road. In addition, some animals that died of vehicle collision are likely to have moved off the road surface after collision (dying later on) and some roadkill was likely removed by scavengers or people.

Our estimates of roadkill frequencies (e.g., per day per km) might be slightly inflated due to the fact that animals found on Mondays could be from any time period after the preceding Friday (given we did not survey Saturday and Sunday). In other words, although we aimed to estimate rates and frequencies per 24 hr, we might have included animals that could have died over the course of multiple days on our counts conducted on Mondays. We did not address this through statistical corrections as that would require us to have more in‐depth knowledge on carcass removal and degradation rates, nor did we include this “Monday factor,” as we wanted to avoid overly complex models.

We highlight the remarkably high number of amphisbaenians *A. bassleri* (62 specimens) that died on the road. Specimens were found on both roads on locations with roadside habitats ranging from disturbed to less disturbed areas during both drier and wetter periods. Little is known about this fossorial animal but our findings confirm previous suggestions that this fossorial species is more abundant than usually expected, and can persist at least temporarily in areas with high levels of habitat alteration (van der Hoek & Jarrín‐V, 2017; Maschio, Santos‐Costa, & Prudente, 2016). Studies of time lags in population responses will be key to explain the true impact of human disturbances on this species in the long term.

Fossorial species are likely to be disproportionally affected by road barriers (Sosa & Schalk, 2016), and we indeed found that the fossorial amphisbaenian *Amphisbaena bassleri* and the semi fossorial snake species *Atractus collaris, Atractus elaps*, and *Atractus major* represented 70% of all reptiles found during this study. However, the majority of these specimens were encountered at a clear hotspot of roadkill of reptiles on a section of road near Ikiam, which requires additional explanation. We propose that the ongoing construction of buildings at the time of sampling and other infrastructure on the campus of Ikiam may have caused disturbances to fossorial species and led to the mass surfacing of these animals. In addition to disturbance from vibrations, fossorial species may have had limited burrowing capabilities in the compacted soil near construction sites (Ducey, Formanowicz Jnr, Boyet, Mailloux, & Nussbaum, 1993).

We collected several reptiles and amphibians (~30 specimens) that presumably died of exhaustion or hyperthermia, and not because of direct collisions with vehicles. We found these specimens usually at the side of the road close to the sidewalk, where there was little chance of runover by vehicles. There are deep gutters, most commonly made of concrete, along the asphalt roads in this area to promote water run‐off during rain. However, these gutters, some of which were up to 30 cm deep, might function as a trap for reptiles and amphibians. Indeed, we made several opportunistic field observations of snakes remaining for prolonged periods of time in these gutters despite seemingly making attempts to escape. We acknowledge some speculation here, but hypothesize that being stuck in these gutters, which are usually without any vegetative cover, overexposes animals to direct solar radiation, the effects of which may be enhanced by heat absorption of the concrete or asphalt. Furthermore, reptiles and amphibians might use the road surface for thermoregulation during the day and or even at night (Shine, Lemaster, Wall, Langkilde, & Mason, 2004; Sullivan, 1981). The combination of these factors with the slow‐moving behavior and elongated body shape (of snakes) may explain the relatively high numbers of roadkill we found for these two taxa.

It is hard to estimate the demographic effects of roadkill on the vertebrate populations because of the lack of data on species abundance in this region. Twenty‐two of the recorded species were not evaluated by the IUCN due to deficient data. The fact that little is known about certain species underlines the need to gain knowledge on these species and the threats that roads may impose on them. Getting insight in abundance data in this area is thus an important but challenging next step for conservation efforts in this region.

Although the majority of the species recorded in this study were common species, a substantial reduction in population size of abundant species could severely alter ecosystem functioning (e.g., affecting productivity, biodiversity, and invasion susceptibility) (Gaston & Fuller, 2007, 2008). Besides, the effects of roads on animal populations may be delayed, and it may take decades to see the effects of roadkill on population level (Andrews & Jochimsen, 2007). As such, we propose that mitigation of roadkill should play a role in the design of new roads in this rapidly developing part of the Neotropics. For this, we need more knowledge of the life histories and ecologies of species of this area. In addition, we need studies that provide recommendations on effective mitigation measures for this region, where measures adapted from other regions of the world might not necessarily function effectively (van der Grift et al., 2013).

In this first study of roadkill in the Amazon region of Ecuador, we found strikingly high numbers of roadkill in a relatively short period, on a short stretch of road. Follow‐up studies should indicate whether this study reflects roadkill patterns in the rest of the Western Amazon. These future studies should also allow us to gather a more complete overview of the impacts of roads on wildlife in the Western Amazon, and indicate the extent and severity of this problem in this hyperdiverse but understudied region of the Neotropics. How we deal with the challenges of development and urban encroachment (Jarrín‐V., Tapia Carrillo, & Zamora, 2017) depends mostly on locally generated information like the one provided in this study.

## CONFLICT OF INTEREST

None declared.

## AUTHOR CONTRIBUTIONS

**Jonathan Filius:** Data curation (lead); formal analysis (lead); methodology (lead); visualization (lead); writing–original draft (lead); writing–review and editing (equal). **Yntze van der Hoek:** Data curation (supporting); formal analysis (supporting); methodology (equal); resources (supporting); Supervision (lead); writing–original draft (supporting); writing–review and editing (equal). **Pablo Jarrín‐V:** Resources (supporting); supervision (supporting); writing–review and editing (supporting). **Pim van Hooft:** Data curation (supporting); resources (supporting); supervision (lead); writing–original draft (supporting); writing–review and editing (supporting).

## Supporting information

Tables S1‐S4

## Data Availability

All data and code related to this manuscript have been archived and are freely available on Dryad (https://doi.org/10.5061/dryad.sqv9s4n1b).
