# Supplementary material for: Wildlife roadkill patterns in a fragmented landscape of the Western Amazon
Source: Ecol Evol. 2020 Jun 20;10(13):6623–35. doi: 10.1002/ece3.6394 (PMC7381557; doi:10.1002/ece3.6394)
Supplement: Supplementary file 1 — Tables S1‐S4 [file ECE3-10-6623-s001.docx]

**Appendix**

| Table S1. Roadkilled species for the E45 and VTMA. Number of roadkilled species, functional group and IUCN status. Functional groups: S (slow), I (intermediate), F (fast) and NA (Not Applicable). Status on the IUCN red list (2017), NE (Not Evaluated), LC (Least Concern). Records indicated with an asterix* were excluded from statistical analyses. | | | | | | | |
| --- | --- | --- | --- | --- | --- | --- | --- |
| Taxon | Common name | N E45 | N VTMA | N Tot | % of taxon | Functional group | IUCN |
| REPTILES |  |  |  |  |  |  |  |
| **Amphisbaena** |  |  |  |  |  |  |  |
| *Amphisbaena fuliginosa bassleri* | Speckled Worm Lizard | 37 | 21 | 58 | 24.5 | S | NE |
| **Serpentes** |  |  |  |  |  |  |  |
| Colubridae |  |  |  |  |  |  |  |
| *Atractus collaris* | Collared Ground Snake | 1 | 28 | 29 | 12.2 | S | LC |
| *Atractus elaps* | Black Ground Snake | 2 | 31 | 33 | 13.9 | S | LC |
| *Atractus major* | Big Ground Snake | 8 | 24 | 32 | 13.5 | S | LC |
| *Chironius fuscus* | Brown Sipo | 2 | - | 2 | 0.8 | S | NE |
| *Chironius multiventris* | South American Sipo | - | 1 | 1 | 0.4 | S | NE |
| *Chironius scurrulus* | Wagler's Sipo | 3 | - | 3 | 1.3 | S | NE |
| *Drymoluber dichrous* | Northern Woodland Racer | - | 1 | 1 | 0.4 | S | NE |
| *Erythrolamprus aesculapii* | Aesculapian False Coral Snake | - | 2 | 2 | 0.8 | S | LC |
| *Erythrolamprus guentheri* | Günther's False Coral Snake | - | 7 | 7 | 3.0 | S | LC |
| *Leptodeira annulata* | Banded Cat-eyed Snake | - | 1 | 1 | 0.4 | S | LC |
| *Mastigodryas boddaerti* | Boddaert's Tropical Racer | 1 | - | 1 | 0.4 | S | NE |
| *Oxyrhopus petolarius digitalis* | Forest Flame Snake | - | 1 | 1 | 0.4 | S | NE |
| *Oxyrhopus vanidicus* | Mimic False Coral Snake | 2 | 1 | 3 | 1.3 | S | NE |
| *Phrynonax polylepis* | Puffing Snake | 3 | 1 | 4 | 1.7 | S | NE |
| *Spilotes sulphureus* | Yellow-bellied Puffing Snake | - | 1 | 1 | 0.4 | S | NE |
| *Tantilla melanocephala* | Black-headed Snake | - | 2 | 2 | 0.8 | S | NE |
| Dipsadidae |  |  |  |  |  |  |  |
| *Clelia clelia* | Mussurana | - | 2 | 2 | 0.8 | S | NE |
| *Dipsas catesbyi* | Catesbyi's Snail Eater | 1 | 4 | 5 | 2.1 | S | LC |
| *Drepanoides anomalus* | Black-collared Snake | - | 2 | 2 | 0.8 | S | NE |
| *Helicops angulatus** | Brown-banded Watersnake | 1 | 7 | 8 | 3.4 | NA | NE |
| *Hydrops martii** | Amazon Water Snake | - | 1 | 1 | 0.4 | NA | NE |
| *Siphlophis compressus* | Tropical Flat Snake | 2 | - | 2 | 0.8 | S | LC |
| *Xenodon rabdocephalus* | False Fer-de-lance | 1 | 5 | 6 | 2.5 | S | NE |
| Elapidae |  |  |  |  |  |  |  |
| *Micrurus lemniscatus helleri* | South American Coral Snake | 1 | 4 | 5 | 2.1 | S | NE |
| *Micrurus ornatissimus* | Ornate Coralsnake | - | 6 | 6 | 2.5 | S | NE |
| *Micrurus narduccii melanotus* | Andean Blackback Coral Snake | 2 | - | 2 | 0.8 | S | NE |
| Unindentified Serpentes* |  |  |  | 16 | 6.8 |  | - |
| **Sauria** |  |  |  |  |  |  |  |
| Teiidae |  |  |  |  |  |  |  |
| *Ameiva ameiva** | Giant Ameiva | 1 | - | 1 | 0.4 | NA | NE |
| Total number of specimen | |  |  | 237 |  |  |  |
| Percentage identified |  |  |  | 93 |  |  |  |
| Species diversity |  | 16 | 22 | 28 |  |  |  |
| AMPHIBIANS |  |  |  |  |  |  |  |
| **Gymnophiona** |  |  |  |  |  |  |  |
| Caeciliidae |  |  |  |  |  |  |  |
| *Caecilia orientalis* | La Bonita Caecilian | 1 | - | 1 | 0.5 | S | LC |
| *Caecilia tentaculata* | Bearded Caecilia | - | 1 | 1 | 0.5 | S | LC |
| Siphonopidae |  |  |  |  |  |  |  |
| *Siphonops annulatus* | Ringed Caecilian | - | 1 | 1 | 0.5 | S | LC |
| **Anura** |  |  |  |  |  |  |  |
| Leptodactylidae |  |  |  |  |  |  |  |
| *Leptodactylus mystaceus* | Basin White-lipped Frog | 2 | 2 | 4 | 2.1 | I | LC |
| *Lithodytes lineatus* | Gold-striped Frog | 4 | 11 | 15 | 7.9 | I | LC |
| Phyllomedusidae |  |  |  |  |  |  |  |
| *Phyllomedusa vaillantii** | White-lined Leaf Frog | - | 5 | 5 | 3.7 | NA | LC |
| Bufonidae |  |  |  |  |  |  |  |
| *Rhaebo ecuadorensis* | - | 26 | 3 | 29 | 15.3 | I | NE |
| *Rhinella dapsilis* | Sharp-nosed Toad | - | - | - | 0.0 | I | LC |
| *Rhinella marina* | Cane Toad | 54 | 28 | 82 | 43.2 | I | LC |
| Hylidae |  |  |  |  |  |  |  |
| *Osteocephalus planiceps** | Flat-headed Bromeliad Treefrog | - | 1 | 1 | 0.5 | NA | LC |
| *Scinax ruber** | Red-Snouted Treefrog | - | 2 | 2 | 1.1 | NA | LC |
| *Trachycephalus macrotis** | Big-eared Milkfrog | - | 1 | 1 | 0.5 | NA | NE |
| Unindentified Anura* |  |  |  | 46 | 24.2 |  | - |
| Total number of specimen | |  |  | 190 |  |  |  |
| Percentage identified |  |  |  | 76 |  |  |  |
| Species diversity |  | 5 | 10 | 12 |  |  |  |
| BIRDS |  |  |  |  |  |  |  |
| **Accipitriformes** |  |  |  |  |  |  |  |
| Cathartidae |  |  |  |  |  |  |  |
| *Coragyps atratus* | Black Vulture | 1 | - | 1 | 1.0 | F | LC |
| Accipitridae |  |  |  |  |  |  |  |
| Unindentified |  | 2 | 1 | 3 | 2.9 | F | - |
| **Apodiformes** |  |  |  |  |  |  |  |
| Trochilidae |  |  |  |  |  |  |  |
| *Amazilia fimbriata* | Glittering-throated Emerald | 1 | 1 | 2 | 2.0 | F | LC |
| *Phaetornis* sp. | Hermit | 1 | 1 | 2 | 2.0 | F | - |
| **Cuculiformes** |  |  |  |  |  |  |  |
| Cuculidae |  |  |  |  |  |  |  |
| *Crotophaga ani* | Smooth-billed Ani | 2 | - | 2 | 2.0 | F | LC |
| **Galliformes** |  |  |  |  |  |  |  |
| Phasianidae |  |  |  |  |  |  |  |
| *Gallus gallus domesticus** | Chicken | - | 2 | 2 | 2.0 | NA | - |
| **Gruiformes** |  |  |  |  |  |  |  |
| Rallidae |  |  |  |  |  |  |  |
| Unindentified* |  | 1 | - | 1 | 1.0 | NA | - |
| **Passiformes** |  |  |  |  |  |  |  |
| Emberizidae |  |  |  |  |  |  |  |
| Unindentified |  | 3 | 1 | 4 | 3.9 | F | - |
| Estrildidae |  |  |  |  |  |  |  |
| *Lonchura* sp. | Munia | - | 1 | 1 | 1.0 | F | - |
| Thraupidae |  |  |  |  |  |  |  |
| *Thraupis episcopus* | Blue-grey Tanager | 6 | 1 | 7 | 6.9 | F | LC |
| *Thraupis palmarum* | Palm Tanager | 1 | - | 1 | 1.0 | F | LC |
| Troglodytidae |  |  |  |  |  |  |  |
| *Troglodytes aedon* | House Wren | 4 | 1 | 5 | 4.9 | F | LC |
| Tyrannidae |  |  |  |  |  |  |  |
| *Myiozetetes similis* | Social Flycatcher | 3 | 3 | 6 | 5.9 | F | LC |
| Unindentified Passiformes | | 40 | 22 | 62 | 60.8 | F | - |
| Unindentified Aves* |  | 3 | - | 3 | 2.9 | NA | - |
| Total number of specimen | |  |  | 102 |  |  |  |
| Percentage identified |  |  |  | 21 |  |  |  |
| Species diversity |  | 11 | 8 | 13 |  |  |  |
| MAMMALS |  |  |  |  |  |  |  |
| **Chiroptera** |  |  |  |  |  |  |  |
| Bat* |  | 9 | 5 | 14 | 21.9 | NA | - |
| **Carnivora** |  |  |  |  |  |  |  |
| Canidae |  |  |  |  |  |  |  |
| *Canis lupus familiaris** | Domestic dog | 3 | - | 3 | 4.7 | NA | - |
| Felidae |  |  |  |  |  |  |  |
| *Felis silvestris catus** | Domestic cat | 1 | 1 | 2 | 3.1 | NA | - |
| **Rodentia** |  |  |  |  |  |  |  |
| Muridae |  |  |  |  |  |  |  |
| Unidentified* |  | 16 | 29 | 45 | 70.3 | NA | - |
| Total number of specimen | |  |  | 64 |  |  |  |
| Percentage identified |  |  |  | 8 |  |  |  |
| Diversity |  | 4 | 3 | 3 |  |  |  |
| Total roadkill |  |  |  | 593 |  |  |  |

| Table S2. Spatial factors affecting the number of roadkill per segment. Models were fitted for each spatial variable separately. AICc, ∆AICc, Standardized regression coefficients (𝛽𝑖) with standard deviation (SE) and *P*–value (*P*) are given for each predictor. Regression coefficients were only standardized for the continuous variables. The relative importance for each model is based on Akaike weights (wAICc) and model fit is given by D^2^. The best supported models are shown in bold. Models where the variables had no significant effect on the number of roadkill per segment are left out. | | | | | | | | |
| --- | --- | --- | --- | --- | --- | --- | --- | --- |
| Functional group | Predictor | AICc | ∆AICc | wAICc | 𝛽_𝑖_ | SE | *P* | D^2^ |
| SLOW | Urban area 100m | 561.64 | 65.83 | 0 | -0.481 | 0.072 | <0.001 | 0.126 |
|  | Urban area 200m | 559.96 | 64.15 | 0 | -0.519 | 0.079 | <0.001 | 0.131 |
|  | Urban area 500m | 559.96 | 64.15 | 0 | -0.519 | 0.079 | <0.001 | 0.131 |
|  | Native forest 100m | 544.23 | 48.42 | 0 | 0.489 | 0.059 | <0.001 | 0.173 |
|  | Native forest 200m | 558.4 | 62.59 | 0 | 0.463 | 0.065 | <0.001 | 0.135 |
|  | Native forest 500m | 532.49 | 36.68 | 0 | 0.642 | 0.077 | <0.001 | 0.204 |
|  | Waterbody 100m | 593.45 | 97.64 | 0 | 0.202 | 0.045 | <0.001 | 0.041 |
|  | Waterbody 200m | 598.44 | 102.63 | 0 | 0.188 | 0.054 | 0.001 | 0.028 |
|  | Shrub 100m | 604.98 | 109.17 | 0 | 0.108 | 0.050 | 0.032 | 0.010 |
|  | Shrub 200m | 605.09 | 109.28 | 0 | 0.108 | 0.052 | 0.036 | 0.010 |
|  | Shrub 500m | 604.89 | 109.08 | 0 | 0.112 | 0.052 | 0.032 | 0.010 |
|  | Distance to water | 587.85 | 92.04 | 0 | -0.376 | 0.090 | <0.001 | 0.056 |
|  | **Distance to RBCC** | **495.81** | **0** | **0.96** | **-0.690** | **0.066** | **<0.001** | **0.303** |
|  | **Distance to city** | **502.34** | **6.53** | **0.04** | **0.652** | **0.063** | **<0.001** | **0.285** |
|  | Number of lanes | 565.68 | 69.86 | 0 | -0.463 | 0.073 | <0.001 | 0.115 |
|  | Speed limit | 604.47 | 108.65 | 0 | 0.006 | 0.003 | 0.040 | 0.011 |
| INTERM-EDIATE | Urban area 100m | 439.14 | 9.89 | 0.01 | -0.369 | 0.087 | <0.001 | 0.0672 |
|  | Urban area 200m | 436.93 | 7.68 | 0.02 | -0.409 | 0.093 | <0.001 | 0.0752 |
|  | Urban area 500m | 436.93 | 7.68 | 0.02 | -0.409 | 0.093 | <0.001 | 0.0752 |
|  | Native forest 100m | 446.49 | 17.24 | 0 | 0.264 | 0.076 | 0.001 | 0.0407 |
|  | Native forest 200m | 440.65 | 11.4 | 0 | 0.336 | 0.080 | <0.001 | 0.0618 |
|  | Native forest 500m | 445.21 | 15.96 | 0 | 0.308 | 0.088 | <0.001 | 0.0454 |
|  | Waterbody 100m | 444.63 | 15.39 | 0 | 0.224 | 0.054 | <0.001 | 0.0474 |
|  | Waterbody 200m | 452.05 | 22.81 | 0 | 0.175 | 0.068 | 0.010 | 0.0207 |
|  | Waterbody 500m | 439.23 | 9.99 | 0.01 | 0.330 | 0.073 | <0.001 | 0.0669 |
|  | Shrub 100m | 436.75 | 7.51 | 0.02 | 0.241 | 0.043 | <0.001 | 0.0758 |
|  | Shrub 200m | 440.45 | 11.21 | 0 | 0.233 | 0.047 | <0.001 | 0.0625 |
|  | Shrub 500m | 445.73 | 16.49 | 0 | 0.206 | 0.051 | <0.001 | 0.0434 |
|  | Distance to water | 452.22 | 22.98 | 0 | -0.223 | 0.100 | 0.026 | 0.0201 |
|  | Distance to RBCC | 453.09 | 23.85 | 0 | -0.178 | 0.081 | 0.028 | 0.0169 |
|  | Distance to city | 453.7 | 24.46 | 0 | 0.166 | 0.081 | 0.040 | 0.0147 |
|  | Number of lanes | 452.52 | 23.27 | 0 | 0.199 | 0.088 | 0.024 | 0.0190 |
|  | **Speed limit** | **429.25** | **0** | **0.92** | **0.022** | **0.004** | **<0.001** | **0.1029** |
| FAST | Cultivated 500m | 347.61 | 19.82 | 0 | -171.77 | 0.155 | 0.028 | 0.0173 |
|  | Number of lanes | 344.11 | 16.32 | 0 | -170.02 | 0.293 | 0.009 | 0.0335 |
|  | **Speed limit** | **327.79** | **0** | **1** | **-161.86** | **0.025** | **<0.001** | **0.1088** |

| Table S3. Factors affecting roadkill distribution, showing all candidate models for each functional group. AICc, ΔAICc, wAICc weights and D^2^ values for every hypothesis are given for the different evaluated scales of 100, 200 and 500 m. The best supported models (ΔAICc < 7) are shown in bold. Abbreviations: Land cover classes; F (native forest), S (shrubland), W (waterbody), U (urban area), C (cultivated land) and P (pasture) and distances; dw (distance to nearest water), dp (distance to RBCC), dc (distance to nearest city) and road characteristics Sp (speed limit) and La (number of lanes). | | | | | | | | | | | | | |
| --- | --- | --- | --- | --- | --- | --- | --- | --- | --- | --- | --- | --- | --- |
|  |  | Slow | | | | Intermediate | | | | Fast | | | |
| Scale | Hypothesis | AICc | ∆AICc | wAICc | D^2^ | AICc | ∆AICc | wAICc | D^2^ | AICc | ∆AICc | wAICc | D^2^ |
|  | Land cover | | | | | | | | | | | | |
| 100 m | F + S + W + U + C + P | 533.33 | 49.11 | 0 | 0.231 | 423.58 | 24.11 | 0 | 0.162 | 386.22 | 44.54 | 0 | 0.016 |
| 200 m | F + S + W + U + C + P | 540.39 | 56.17 | 0 | 0.194 | 420.07 | 20.59 | 0 | 0.151 | 381.24 | 39.55 | 0 | 0.009 |
| 500 m | F + S + W + U + C + P | 550.16 | 65.94 | 0 | 0.186 | 427.68 | 28.2 | 0 | 0.147 | 384.47 | 42.79 | 0 | 0.024 |
| 100 m | F + S + W | 552.51 | 68.29 | 0 | 0.162 | 421.8 | 22.33 | 0 | 0.145 | 380.15 | 38.46 | 0 | 0.014 |
| 200 m | F + S + W | 506.54 | 22.33 | 0 | 0.303 | 419.05 | 19.58 | 0 | 0.178 | 369.79 | 28.11 | 0 | 0.092 |
| 500 m | F + S + W | 526.37 | 42.15 | 0 | 0.232 | 417.34 | 17.87 | 0 | 0.161 | 380.24 | 38.56 | 0 | 0.013 |
|  | Distance | | | | | | | | | | | | |
| NA | dp + dc | 495.15 | 10.93 | 0 | 0.310 | 455.08 | 55.61 | 0 | 0.017 | 380.27 | 38.59 | 0 | 0.003 |
| NA | dw + dp | 497.8 | 13.59 | 0 | 0.303 | 452.67 | 53.2 | 0 | 0.026 | 380 | 38.31 | 0 | 0.014 |
| NA | dw + dp + dc | 497.1 | 12.88 | 0 | 0.310 | 454.14 | 54.66 | 0 | 0.028 | 377.94 | 36.25 | 0 | 0.014 |
|  | Road characteristics |  | | | | | | | | | | | |
| NA | Sp + La | 563.07 | 78.85 | 0 | 0.128 | 427.38 | 27.9 | 0 | 0.117 | **342.01** | **0.33** | **0.33** | **0.182** |
|  | Road characteristics + Land cover | | |  |  |  |  |  |  |  |  |  |  |
| 100 m | Sp + La + F + S + W | 517.34 | 33.13 | 0 | 0.268 | 408.7 | 9.23 | 0.01 | 0.207 | **343.01** | **1.33** | **0.2** | **0.207** |
| 200 m | Sp + La + F + S + W | 530.34 | 46.13 | 0 | 0.233 | 410.48 | 11.01 | 0 | 0.201 | **341.68** | **0** | **0.38** | **0.214** |
| 500 m | Sp + La + F + S + W | 520.1 | 35.88 | 0 | 0.260 | **399.47** | **0** | **0.96** | **0.241** | **344.79** | **3.1** | **0.08** | **0.199** |
|  | Full | | | | | | | | | | | | |
| 100 m | F + S + W + U + C + P + dw + dp + dc + Sp + La | **485.2** | **0.98** | **0.38** | **0.390** | 412.45 | 12.98 | 0 | 0.243 | 350.92 | 9.23 | 0 | 0.234 |
| 200 m | F + S + W + U + C + P + dw + dp + dc + Sp + La | 495.71 | 11.5 | 0 | 0.362 | 416.08 | 16.6 | 0 | 0.230 | 349.97 | 8.28 | 0.01 | 0.238 |
| 500 m | F + S + W + U + C + P + dw + dp + dc + Sp + La | **484.22** | **0** | **0.62** | **0.393** | **406.45** | **6.98** | **0.03** | **0.265** | 352.44 | 10.75 | 0 | 0.227 |

| Table S4. Similarity matrix. Spearman correlations (Spearman’s ρ) between every possible pair of variables for  the 100, 200 and 500 m buffers. | | | | | | |
| --- | --- | --- | --- | --- | --- | --- |
|  | Urban_area_  100m | Native_forest_  100m | Waterbody_  100m | Cultivated_  100m | Pasture_  100m | Shrub_  100m |
| Urban_area_100m | 1 | 0.55 | 0.08 | 0 | 0.27 | 0.05 |
| Native_forest_100m |  | 1 | 0.1 | 0.02 | 0 | 0 |
| Waterbody_100m |  |  | 1 | 0 | 0.02 | 0 |
| Cultivated_100m |  |  |  | 1 | 0 | 0.04 |
| Pasture_100m |  |  |  |  | 1 | 0 |
| Shrub_100m |  |  |  |  |  | 1 |
|  | Urban_area_  200m | Native_forest_  200m | Waterbody_  200m | Cultivated_  200m | Pasture_  200m | Shrub_  200m |
| Urban_area_200m | 1 | 0.61 | 0.08 | 0 | 0.23 | 0.04 |
| Native_forest_200m |  | 1 | 0.08 | 0 | 0 | 0 |
| Waterbody_200m |  |  | 1 | 0 | 0.01 | 0 |
| Cultivated_200m |  |  |  | 1 | 0 | 0 |
| Pasture_200m |  |  |  |  | 1 | 0 |
| Shrub_200m |  |  |  |  |  | 1 |
|  | Urban_area_  500m | Native_forest_  500m | Waterbody_  500m | Cultivated_  500m | Pasture_  500m | Shrub_  500m |
| Urban_area_500m | 1 | 0.49 | 0.03 | 0.03 | 0.15 | 0.08 |
| Native_forest_500m |  | 1 | 0.01 | 0 | 0.01 | 0 |
| Waterbody_500m |  |  | 1 | 0 | 0.03 | 0.02 |
| Cultivated_500m |  |  |  | 1 | 0.01 | 0.01 |
| Pasture_500m |  |  |  |  | 1 | 0.03 |
| Shrub_500m |  |  |  |  |  | 1 |
|  | Dist_city | Dist_water | Dist_park | Speed_limit | Lanes |  |
| Urban_area_100m | 0.25 | 0.15 | 0.26 | 0.28 | 0.07 |  |
| Native_forest_100m | 0.2 | 0.29 | 0.35 | 0.14 | 0.11 |  |
| Waterbody_100m | 0.12 | 0.21 | 0.11 | 0.04 | 0.02 |  |
| Cultivated_100m | 0.01 | 0.04 | 0 | 0.02 | 0.01 |  |
| Pasture_100m | 0.03 | 0.01 | 0.01 | 0.08 | 0.01 |  |
| Shrub_100m | 0.04 | 0.01 | 0 | 0.05 | 0.02 |  |
| Urban_area_200m | 0.23 | 0.19 | 0.26 | 0.28 | 0.08 |  |
| Native_forest_200m | 0.2 | 0.3 | 0.34 | 0.15 | 0.11 |  |
| Waterbody_200m | 0.23 | 0.22 | 0.21 | 0.02 | 0.05 |  |
| Cultivated_200m | 0.01 | 0 | 0 | 0.02 | 0.01 |  |
| Pasture_200m | 0.02 | 0 | 0.01 | 0.09 | 0.02 |  |
| Shrub_200m | 0.05 | 0.01 | 0 | 0.04 | 0.02 |  |
| Urban_area_500m | 0.25 | 0.13 | 0.35 | 0.11 | 0.15 |  |
| Native_forest_500m | 0.21 | 0.23 | 0.42 | 0.09 | 0.22 |  |
| Waterbody_500m | 0 | 0.05 | 0.07 | 0 | 0.01 |  |
| Cultivated_500m | 0 | 0 | 0.01 | 0 | 0 |  |
| Pasture_500m | 0.03 | 0.01 | 0 | 0.06 | 0 |  |
| Shrub_500m | 0.03 | 0.01 | 0.02 | 0.03 | 0 |  |
| Dist_city | 1 | 0.12 | 0.61 | 0.06 | 0.16 |  |
| Dist_water |  | 1 | 0.22 | 0.08 | 0.1 |  |
| Dist_park |  |  | 1 | 0.06 | 0.48 |  |
| Speed_limit |  |  |  | 1 | 0.03 |  |
| Lanes |  |  |  |  | 1 |  |
